# Immunoglobulin sub-class distribution in bipolar disorder and schizophrenia: potential relationship with latent *Toxoplasma Gondii* infection

**DOI:** 10.1186/s12888-018-1821-9

**Published:** 2018-07-27

**Authors:** Nora Hamdani, Djaouida Bengoufa, Ophélia Godin, Raphaël Doukhan, Emmanuel Le Guen, Claire Daban-Huard, Meriem Bennabi, Marine Delavest, Jean-Pierre Lépine, Wahid Boukouaci, Hakim Laouamri, Josselin Houenou, Stéphane Jamain, Jean-Romain Richard, Philippe Lecorvosier, Robert Yolken, Krishnamoorthy Rajagopal, Marion Leboyer, Ryad Tamouza

**Affiliations:** 1 Inserm U955, Team 15 «Genetic Psychiatry », F-94000 Creteil, France; 2 AP-HP, DHU Pe-PSY, Paris Est Créteil University, Henri Mondor - Albert Chenevier, Group, psyChiatry, F-94000 Creteil, France; 3 Fondation Fondamental, Scientific Cooperation Foundation, F-94010 Creteil, France; 4 CEA Saclay, Neurospin, Gif-Sur-Yvette, France; 5 AP-HP, Paris Diderot University, Psychiatry, Lariboisiere Fernand Widal Hospital, F-75010 Paris, France; 6 CIC 006Henri Mondor INSERM & Biological Resource Platform, Paris Est University, AP-HP, Creteil, France; 7 Jean Dausset Laboratory, LabEx Transplantex & INSERM, UMRS 1160 Saint Louis Hospital, Paris Diderot University, F75010 Paris, France; 8 Stanley Laboratory of Developmental Neurovirology, Johns Hopkins University Medical Center, Baltimore, USA

**Keywords:** Humoral immunity, Immunoglobulins, Bipolar disorder, Schizophrenia, *Toxoplasma gondii*

## Abstract

**Background:**

Immune dysfunction could play a significant role in the pathogenesis of bipolar disorder (BD) and schizophrenia (SZ), conditions with an underlying pro-inflammatory state. Studies on humoral immune responses (which reflects antibody mediated fight against pathogens) in schizophrenia and bipolar disorder are sparse and often providing contradictory results. The aim of this study was to assess humoral immunity in a group of stable bipolar disorder and schizophrenia patients compared to controls by determining total Immunoglobulins and IgG subclasses and to assess their association with latent *Toxoplasma gondii* and/or CMV infection.

**Methods:**

334 subjects (124 BD, 75 SZ and 135 Healthy Controls [HC]) were included and tested for humoral immunity by determining the total immunoglobulins (IgG,A and M) and IgG subclasses (IgG1, IgG2, IgG3, IgG4) and their relationship with latent *Toxoplasma gondii* infection, an established risk factor for BD and SZ.

**Results:**

Although lower levels of IgG, IgG1, IgG2, IgG4 and IgA were found among BD as compared to HC and/or SZ, after adjustment for confounding variables, only low levels of IgG and IgG1 in BD remai- ned significant. Strikingly highest levels of antibodies to T. gondii (but not CMV) infection in BD and SZ were associated with lowest levels of IgG3 and IgG4 levels as compared to controls.

**Conclusions:**

Schizophrenia and bipolar disorder patients with latent T. *gondii* specific infection may be more vulnerable to changes in immuno-inflammatory processes than controls with similar latent infectious state. Simultaneous sequential immunological monitoring both in steady state and active disease phases in the same BD and SZ patients are warranted to understand the role of *Toxoplasma gondii* latency in these disorders.

## Background

There is a growing body of evidence that immune dysfunction could play a significant role in the pathogenesis of bipolar disorder (BD) and schizophrenia (SZ). Indeed, signs of inflammation and immune dysfunctions have consistently been observed in both disorders. In BD, both manic and depressive episodes have been associated with pro-inflammatory states with involvement of innate and adaptative immune systems [12, 28]. Indeed, in acute phases of the disorder, exacerbated stimulation of Th1 cells (mediator of cellular immune response) and enhanced production of pro-inflammatory cytokines are reported. In SZ, an imbalanced Th1/Th2 ratio has consistently been observed with a blunted type1 response (decreased levels of interferon gamma (IFN- g), Interleukin-2 (IL-2), and soluble IL-2 receptors (sIL-2R)) along with raised levels of type 2 cytokines viz IL-6 and IL10 [17, 20–22]. Besides, phases of depression/mania and SZ are often accompanied by an inflammatory surge, as evidenced by expression of acute phase proteins in such situations [12, 21, 22].

So far, studies on humoral immune responses in major psychiatric disorders are sparse, of small size, and often providing contradictory results. In BD, one study described an elevation of IgG while another found no change [29, 35]. A different study showed elevated levels of IgM in BD while Tudorache et al. found rather a decrease in IgM levels in this disorder [15, 34]. Yet another report described a decreased IgD levels in BD [35]. Studies focusing on SZ revealed an elevation of IgA, IgM or IgG in the patients’ sera or CSF during acute phases of the disease ([1, 3, 6, 10, 11, 16, 27, 29, 32]). Interestingly, in SZ, IgG raises gradually to reach a peak at about 12 to 17 years after the onset typically mirroring persistence of an underlying infection [10, 33]. This study highlighted the influence of the disease state on temporal variations of the measured biological parameters and may partly explain the reported inconsistencies among studies. Additional but important confounder is the environmentally acquired infections that can influence or trigger the intercurrent inflammatory/immune responses.

Lines of arguments for the role of infections (especially when contracted during pregnancy or perinatally) in the etiology of SZ and BD have accumulated over years through large epidemiological studies. Strikingly the skewed birth peak of SZ and BD during winter season may be corollary to infectious epidemics (eg: influenza) prevalent in that season. Many other infectious agents viz CMV (cytomegalovirus), Herpes and *Toxoplasma gondii,* known for their CNS tropism (when contracted during pregnancy), have been shown to be the risk factors for SZ and BD [4, 5, 7, 8, 12, 19, 23, 31, 36]. Actually, the mechanism by which *T. gondii* and viruses constitute risk factors for schizophrenia and bipolar disorder have not been elucidated. It should be interested to assess the humoral immunity known to be altered in both disease and assess their link with latent infection to T. gonddi and/or CMV.

Given the above described inconsistencies in the data on humoral response in BD and SZ and paucity of information on patients at stable state, we undertook this study:

- to profile the humoral immunity in a group of stable BD and SZ patients by determining total immunoglobulins and IgG subclasses
- to assess its relationship with latent T. *gondii* and or/CMV infection

## Methods

Patients (both in and outpatients) with BD (type I and II), and SZ meeting the DSM-IV criteria [2], consecutively admitted/consulted at two French university-affiliated psychiatric departments, (Henri Mondor hospital, Créteil, University of Paris-East and Fernand Widal hospital, Paris, University of Diderot, Paris), were included in the present study after approval by the institutional ethical committee and obtention of written informed consent from the participants. Both BD and SZ were recruited at steady state with scores YMRS< 8 and MADRS< 12 for the former and PANSS< 60 for the latter. Healthy controls (HC) were enrolled through a clinical investigation center, also in Paris, France (Center for Biological Resources, Henri Mondor hospital, Créteil, France). Only those without a personal or first degree family history of any psychotic or affective disorders, addictive or suicidal behavior, as measured by the Family Interview for Genetic Studies (FIGS) [24] and a personal or family history of autoimmune diseases (information obtained by self-report or from medical records) were included. Other exclusion criteria included i) current or past immunosuppressive treatment ii) recent infection or an ongoing inflammatory disease iii) a positive serology for HIV1/2; Hepatitis A, B and C prior to enrollment iv) neurological disorder with cognitive impairment. The age range of both patients and controls were between 18 and 65 years.

Patients were interviewed with a French version of the “Diagnostic Interview for Genetic Studies” (DIGS, 1994, [25, 26]) for the assessment of lifetime clinical characteristics of BD SZ as well as for demographic characteristics (i.e. education level, working status, season of birth, birth place/country). Current medications as well as hospitalization status were recorded. At enrollment, manic symptoms were assessed with the Young Mania Rating Scale [38] and depressive symptoms with the Montgomery and Asberg Depression Rating Scale [18] for BD. Both SZ and BD participants were further evaluated using the Positive and Negative Syndrome Scale (PANSS) [14]. Nicotine dependence was assessed using the Fagerström scale [13] and recent or past alcohol or drug abuse were recorded for all the participants.

### Serological testing for immunoglobulins

Total IgG, IgA, and IgM were quantified by immunoturbidimetry using commercially available immunoassay reagents (COBAS). IgG sub-classes i.e. IgG1, IgG2, IgG3 and IgG4 levels were determined on a SPAPLUS analyzer (The Binding Site, Birmingham, U.K.) using commercially available kits (The Binding Site, Birmingham, U.K.).

### *Serological testing for T. gondii exposure* and CMV

On enrollment solid- phase enzyme immunoassay was performed to assess the IgG, IgM, and IgA antibodies against *Toxoplasma gondii (T.Gondii)* as previously described [7]. Plasma was separated from the blood sample by centrifugation and stored at − 70 until testing. After testing, the sample was thawed and test for IgG class antibodies to *Toxoplasma gondii* using solid phase enzyme immunoassay as previously described [37]. Assay reagents were obtained from IBL America, Minneapolis Minn. A standard sample with a known amount of antibody was also analyzed in each assay run. For each antibody measurement, a sample was considered to be seropositive for *T. gondii* if it generated a signal greater than 80% of the mean signal generated by a positive standard provided in the test kit [9], corresponding to approximately 10 International Units (IUs).

Solid phase immunoassay techniques were used to measure IgG class antibodies to Cytomegalovirus in the sera of all participants as previously described [7].

### Statistical analyses

Analyses were performed for data from 334 participants. Distribution profile of the quantitative variables was analysed by Shapiro-Wilk statistics. Standard descriptive analysis (X2 for categorial variables, Wil- coxon-Mann-Whitney test for association with non-normal continuous variables and t-test for association with continuous variables) were performed to compare patients and controls two by two for socio- demographic and clinical variables as well for infectious status of T. gondii. To evaluate the multivariate relationship of immunoglobulin levels in patients and controls, analysis of covariances adjusted for age, gender, tobacco and BMI were used. Additional adjustment for disease duration was made to compare BD and SZ patients. In order to evaluate the association between IgG levels and T. *gondii* seroinfection, IgG levels were dichotomized as first quartile (lowest levels) versus the rest of the distribution and the two groups were compared in terms of T. *gondii* antibodies levels. Analyses were performed in the all sample and in sub- group: controls and patients together (BD + SZ).

For the independent samples, T-test, Cohen’s d was determined by calculating the mean ddiffrence between groups, and then divided by the pooled standard deviation.

Tests were two-sided and significance level was fixed at 5%. All analyses were conducted using SAS (release 9.3; SAS Statistical Institute, Cary, NC [30]).

## Results

### Demographic and clinical variables

Demographic, clinical and biological variables of the study participants (124 BD, 75 SZ and 135 HC) are detailed in Table 1. A majority of them are chronic patients and at enrollment they were in stable phase.

**Table 1:** Sociodemographic, clinical, serologic, and cognitive variables between bipolar disorder, schizophtrenic patients and healthy controls

Variables	BD (*n* = 124)	Statistical test, p	SZ (*n* = 75)	Statistical test value, p	HC (*n* = 135)	Statistical test, p
		BD vs C		SZ vs C		BD vs SZ
Number of participants (BD/SZ/HC): 334	124		75		135	
*Sociodemographic and clinical variables*						
Age (in years)(mean ± sd)	44,2 (13,5)	*0.0017*	36,6 (11,9)	0.0858	39,5 (13,7)	< 0,0001
Gender (%female)	52.8	0.2469	28	< 0,0001	60	0.0006
Caucasian (%yes/no)	86	< 0,0001	73	0.1006	60.8	0.0367
Educational level (%high school)	57.6	0.0111	28.6	0.0059	43.9	< 0,0001
Married (%yes)	34.8	0.0092	79.5	< 0,0001	48.7	< 0,0001
Birth place (%urban)	87.4	0.5936	94	0.1599	89.3	0.0723
Childhood upbringing(urban) (%yes)	84	0.1514	87.9	0.7006	89.4	0.3705
Smoker (%yes)	62.6	< 0,0001	64	< 0,0001	25.4	0.8432
BMI (mean ± sd)	25,1 (4,2)	0.0169	25,6 (5,5)	0.0398	24,2 (4,1)	0.9519
Age at onset (mean ± sd)	26,5 (10,6)		23,4 (7,6)			0.0084
Number of total episode (mean ± sd)	8,0 (6,8)		3,93 (3,3)			
Duration of the disease (years) (mean ± sd)	17,5 (12,5)		12,6 (10,5)			0.0006
Auto-immune disease (%)	12%		16.20%		0	0.1726
Childhood infections (%positive)						
MADRS	7,25 (8,8)		9,25 (7,5)			
YMRS	5,5 (7,5)		6,3 (6,1)			
PANSS positive	8,9 (4,6)		16,2 (6,6)			
PANSS negative	9,4 (4,5)		20,2 (8,4)			
PANSS general	22,3 (9,8)		33,7 (11,0)			
PANSS total score	39,8 (16,1)		68,3 (22,1)			
*Serologic variables*						
T. gondii (IgG, %positive)	76.2	0.0006	68	0.1048	58.2	0.1501
Co-infection (IT gondii + CMV, %positive)	47.2	0.291	35.6	0.4826	40.6	0.1146

Significant level was fixed at *p*<0.05

“All of them were under medications; BD patients received either lithium (35%) or anticonvulsants (27%) or atypical antipsychotics (4%) alone or a combination of two mood stabilizers (9%) or a combination of a mood stabilizer with an atypical antipsychotic (22%). Patients with SZ received either an atypical antipsychotic (68%) or a typical antipsychotic (9%) or a combination of a mood stabilizer with an atypical antipsychotic (20%) or two antipsychotics (6%)”.

### Toxoplasma gondii status

A total of 94 (76.2%) BD, 51 SZ (68%) and 79 HC (58.2%) were IgG seropositive for *T. gondii* with a significant difference between BD and HC (Table 1). None of the participants was in acute phase of *T. gondii* infection.

### Total immunoglobulins and IgG subclasses

Significantly lower levels of IgG were found in BD as compared to HC (*p* < 0, 0001) and SZ (p < 0, 0001) (Table 2). Concerning the subclasses of IgG, only IgG1 level in BD differed significantly from that of SZ and HC after adjustement for confouding variables (Table 2). When duration of the disease is taken into consideration, both the total IgG and IgG1 levels were lowest for the BD group. No significant difference was noted in total IgA and IgM levels among BD, SZ and HC.

**Table 2:** Immunoglobulin in bipolar disorder, schizophtrenia and healthy controls

Variables	BD (*n* = 124)	Effet size*	Statistical test, p	p adjusted for age, gender, tobacco and BMI	SZ (*n* = 75)	Effet size*	Statistical test value, p	p adjusted for age, gender, tobacco and BMI	HC (*n* = 135)	Effet size^a^	Statistical test, p	p adjusted for age, gender, tobacco, BMI and duration of the disease
		BD vs C				SZ vs C				BD vs SZ		
Number of participants (BD/SZ/HC):	124				75				135			
Immunoglobulins (g/l)(mean ± sd)												
IgG	10,3 (2,2)	d = 0,72	< 0,0001	0.0016	12,1 (3,4)		0.477		12,1 (2,5)	d = 0,64	< 0,0001	0.0002
IgG1	5,4 (1,6)	d = 0,60	< 0,0001	0.0076	6,8 (2,2)		0.2998		6,4 (1,7)	d = 0,74	< 0,0001	< 0,0001
IgG2	3,6 (1,4)	d = 0,43	0.0003	0.407	3,7 (1,3)	d = 0,36	0.0102	0.9952	4,2 (1,4)		0.4529	
IgG3	703,9 (368,4)		0.1736		706,3 (377,1)		0.2209		783,1 (468,4)		0.9726	
IgG4	332,3 (310,8)		0.1958		465,1 (359,6)		0.0747		388,4 (345,7)	d = 0,40	0.0046	0.161
IgA	2,0 (0,7)	d = 0,25	0.0185	0.111	2,1 (0,9)		0.136		2,2 (0,9)		0.8435	
IgM	1,0 (0,5)		0.2688		1,0 (0,5)		0.102		1,1 (0,6)		0.4491	

^a^Effect size wad determined using the Cohen’s d coefficient by calculating the mean difference between groups, and then divided by the pooled standard deviation

### Relationship between IgG levels and T. Gondii seroinfection

We found that highest levels of antibodies to T. *gondii* infection in BD and SZ were associated with lowest levels of IgG3 (3.1 ± 1.8, vs 2.6 ± 1.8, *p* = 0.03) and IgG4 levels (3.1 ± 1.8 vs 2.6 ± 1.8, *p* = 0.04) as compared to controls.

Conversely, we did not find such an association with viruses such as HSV1&2 and CMV.

## Discussion

In the present study, we found that levels of IgG and IgG1, were lower among BD as compared to SZ and HC after correcting for confounding factors (tobacco use, age, gender and BMI) and may suggest that the decrease in the total IgG levels in our patients may be due to a significant decrease in the production of IgG1. We also found that IgG2 and IgA were lower in BD compared to HC and that IgG2 were lower in the SZ group as compared to HC but did not remain after corrections for confounding factors. We also found that lowest levels of IgG3 and IgG4 were associated with highest antibodies to T. *gondii* in BD and SZ as compared to controls. Interestingly, our date could not be explained by a co-infection to viruses such as CMV. Our study, essentially focusing on stable phase of BD and SZ, provide data that differ significantly from published reports. First, Sane et al. [29] reported in affective disorders, patients had high levels of total IgG. But this study involved a small number of patients in acute phase at enrollment (*n* = 16) as compared to the present work (*n* = 124). Further the authors failed to take into account the confounding factors. Another study linking Ig levels to mania (with corrections for the influence of age and gender), revealed elevated levels of both total IgG and IgG1, suggesting that acute manic state may be accompanied by an inflammatory response [35]. It is recognized that acute phase (both in mania and to a lesser extent in depression), is characterized by an inflammatory surge when moving from euthymic state [12].

The low levels of IgG and IgG1 observed here can mark the stable phase of the disease and/or related to the duration of the disease. For example, in SZ during acute crisis and also depending upon the duration of the disease, an increase in the mean serum level of total IgG was observed [1, 3, 6, 10, 11, 27, 29, 32]. In the present study, as the low levels of IgG and IgG1 persisted in the BD group when the duration of the disease is taken into consideration, One may hypothesize that at stable phase, the inflammatory processes might be less vigorous than in acute phase of established infection, say T. gondii or other intercurrent infections from environmental sources may contribute to such inflammatory flare up in BD and SZ. Additional possibility is that intercurrent infections alter the immuno-inflammatory system such that in susceptible (with latent infectious state) BD and SZ patients, humoral response from memory B cells are activated.

The finding that low levels of total IgG3 and IgG4 were associated with high levels of T. *gondii*-specific antibodies may lend some support to such possibility. In other words, both SZ and BD patients with latent T. *gondii* specific infection may be more vulnerable to changes in immuno-inflammatory processes than controls with similar latent infectious state. However, Toxoplasma seroprevalence in each group is limited by the small numbers of patients and controls. Besides, because of the small sample size of our study, we failed to find a significant difference between schizophrenia and healthy controls regarding the seroprevalence of T. gondii.

The limitation of our study resides with the fact that we did not perform simultaneous sequential immunological monitoring both in steady and active disease phase in the same BD and SZ patients. Furthermore, patients and controls were not matched for age and gender, which could influence our data. Such studies are mandatory not only to get further insight into the impact of T. *gondii* latency but also other well-defined infectious risk factors in these major psychiatric disorders.
